# Preventive Health Behaviours among Adolescents and Their Parents during the COVID-19 Outbreak in the Light of the Health Beliefs Model

**DOI:** 10.3390/ijerph192417060

**Published:** 2022-12-19

**Authors:** Marietta Koźlarek, Natalia Błaszczyk, Magdalena Grajek, Sylwia Jaskulska

**Affiliations:** 1 Faculty of Physic, Adam Mickiewicz University, 61-712 Poznan, Poland; 2 Faculty of Physic, University of Warsaw, 00-927 Warsaw, Poland; 3 Faculty of Educational Studies, Adam Mickiewicz University, 61-712 Poznan, Poland

**Keywords:** preventive health behaviours, COVID-19 outbreak, adolescents, parents, Health Beliefs Model, cue to action

## Abstract

This article analysed the relationship between the preventive health behaviours of parents and teenagers during the COVID-19 outbreak, taking the Health Beliefs Model (HBM) as a point of reference. We assumed that parents’ behaviours may be a cue to action for adolescents, looking at their preventive health behaviours regarding vaccination against COVID-19, as well as vaccination intention (among unvaccinated people); wearing protective masks where it is compulsory and where it is not obligatory; and maintaining physical distance and disinfecting hands in public places. The collected data were statistically analysed using the Statistica version 13.3 software package for advanced statistical data analysis. Descriptive statistics and correlation for non-parametric data (Spearman’s correlation) were used. Research on a sample of 201 parents and their children revealed that young people engage in preventive behaviour less frequently than parents, but that the likelihood of such behaviour increases if they have a parent’s cue to action. When formulating recommendations, we considered the gender of the surveyed parents, as the questionnaire was mainly completed by women, which may be an indicator of the unequal involvement in addressing the topic of the pandemic and preventive health behaviours, including attitudes towards vaccines.

## 1. Introduction

As a result of the COVID-19 pandemic, the everyday life of Poles has changed. Restrictions and recommendations were introduced to minimize the virus spread, which were mainly concerned with limiting social contact (i.e., temporary transition of schools, universities and companies to distance work mode; prohibitions related to the organization of events; exclusion from the use of public places; closure of service points; and even restrictions on movement), regulations concerning hygiene-related behaviour (wearing protective masks, disinfecting hands and surfaces) and introducing the possibility of vaccinations [1]. The vaccination programme in Poland started on 27 December 2020, and the following groups were initially included: medical staff, employees of social welfare homes and municipal social welfare centres, people employed at medical universities, students of medical faculties and teachers. All Poles were then included in the programme, starting with the oldest, and the last groups included were youth and children from 5 years of age. After a few months, vaccination with booster doses was initiated. Vaccinations were voluntary and free of charge throughout. Vaccines from different companies were used. Starting with the third dose, patients could choose which vaccine was used [2,3]. These restrictions and recommendations influenced how tasks related to professional work, family life and forms of spending free time were carried out [4,5]. A positive attitude towards preventive actions was more characteristic of the older rather than the younger population, as well as among people with higher education and women more than men [6,7,8].

This paper analyses the relationship between preventive health behaviours during the COVID-19 outbreak taken by parents and their teenage children. We analyse these relationships and determinants considering the assumptions of the Health Beliefs Model (HBM). The model was originally created as an approach to finding an explanation in the case of problems with participation in preventive or disease-detecting programmes [9]. HBM defines the circumstances that change people’s attitudes towards taking such actions and, therefore, the circumstances that should be taken into account when designing them. The model thus has an educational character. HBM indicates that people are most likely to take preventive action if they perceive the health risks as serious, feel personally vulnerable, if involvement is expected to have more significant benefits than costs and/or when there are some cues that trigger actions (cue-to-action) in their environment [10]. It means that if a person perceives a threat to their health, the perceived benefits of health behaviours outweigh the barriers and there are some circumstances that trigger actions (e.g., education, media information, important persons behaviours), they are more likely to take the recommended preventive health actions [11].

Considering the assumptions of the HBM, many studies have been conducted in education and public health, particularly on the effective prevention of risky behaviours and health education [12,13,14,15,16]. The model can explain, for example, global trends pointing to age as a factor differentiating health behaviours during the COVID-19 pandemic. Adolescents are generally poorly prepared to assess the potential risks of diseases properly, which leads to their strong belief that they cannot become infected [17]. This distorted thinking can lead to a disregard for health advice and exposure to disruptive behaviour that increases the risk of infection [18].

During the COVID-19 pandemic, there was also a narrative that young people were not threatened by the consequences of falling ill. There were calls addressed to adolescents for responsible behaviour, but only to protect the health and safety of others (the elderly) [19,20,21]. Thus, it can be assumed that neither a personal sense of threat nor a sense of personal benefits was created, and certainly not one strong enough to change the behaviour of young people.

The HBM assumes several possible ways of changing attitudes: increasing the perception of benefits, reducing the perception of barriers to effective prevention, increasing the perception of self-efficacy and receiving a cue to action [22,23]. The last path, in the case of young people, can be represented by family and school influences, which are essential elements in changing attitudes [11]. Parents play a significant role in preventing risky health behaviours during adolescence. As the research results show, health behaviours learned at home are difficult to change in later years [24,25,26]. Parents can thus shape their children’s positive health attitudes by providing a cue to action [27,28]. For example, in a study by Neumark-Sztainer et al. [28], one of the two factors most strongly associated with changes in adolescents’ physical activity was the support of peers, parents and teachers for this activity. Family patterns are also a protective factor against risky sexual behaviours [29]. During the COVID-19 pandemic, the relationship between family factors and health behaviours undertaken by adolescents was also indicated. For example, in a study by Wong et al. [30], exchanging information on COVID-19 with family members mediated between individual health awareness and personal preventive behaviour. This relationship also applies to vaccination. As Chen et al. [31] have demonstrated, the willingness of parents and guardians to vaccinate themselves was a potentially significant predictor of children’s vaccination.

The main research question is: What is the relationship between parents’ and teenagers’ preventive health behaviours during the COVID-19 outbreak and its conditions? Taking the HBM as a reference, we assume that the preventive health behaviours of parents, including the fact that they are vaccinated or are about to be vaccinated, may be related to adolescents’ health behaviours as a cue to action, balancing—or at least mitigating—initial beliefs. As Strecher and Rosenstock [9] have postulated, cue-to-action is a little studied phenomenon, and although its stated influence on behaviour is great, there is a need to study it to explain its triggering mechanism.

We assume that, during the COVID-19 pandemic, young people did not meet the assumptions of the HBM in their preventive choices, such as perceiving the health risks as serious, feeling personally vulnerable and expecting to have more significant benefits than costs; thus, in their case, getting cues-to-action could be an important trigger for preventative behaviours. Analyses provided in this direction can improve understanding of the mechanisms affecting the preventive behavioural decisions and provide guidance for practice, for example, in the field of parental pedagogy.

## 2. Materials and Methods

### 2.1. Participants

The selection of the study group was intentional: the questionnaire was addressed to public high school students and their parents. The exclusion criteria were as follows: lack of a complete set answers of student and parent or attending a non-public school. The study was conducted using a Google spreadsheet that respondents received from the head of the educational institution. Only complete data sets were qualified for the study, correlated with the password set by the parents. We obtained 201 such sets: 201 adolescents aged 14 to 17 studying in secondary schools and 201 people aged 34 to 71 being their parents. Considering the population of Poland in 2021, we determined the sample to be representative. Thus, with a fraction size = 0.5 (50%), a maximum error of 5% and a confidence level = 95% (α = 0.95), the minimum sample size was identified as 384 people [32]. After crossing the identified minimum number of data qualified for the study, the survey data collection was finalized to avoid collecting data at different timeslots. This decision was supported by the fact that the situation in Poland, the attitude to vaccination and so on were changing dynamically, and we wanted to collect data in very similar conditions. The questionnaire was active for only a few days in October 2021. In the case of the number of high school students in schoolyear 2020/2021 [33], for complete data student–parent sets (201 pairs), the confidence level is 84% (α = 0.84) (the minimum sample size for students is 197). The study group of students was balanced in terms of basic characteristics, such as gender (40.8% of the respondents were girls, 57.21% boys). Teenagers attended schools located in villages (33.83%), small towns (37.81%) and large cities (28.36%) (Table 1).

The study group of parents was also balanced in terms of place of residence: adults lived in villages (35.32%), small towns (35.82%) and large cities (28.86%). The situation was different for gender, however; 93.03% of the surveyed adults were female, and only 6.97% were male (Table 2).

### 2.2. Procedure

We analysed the relationship between parents’ and teenagers’ preventive health behaviours during the COVID-19 outbreak and connected factors. The respondents were informed about the purpose of the research, who it was directed towards, as well as about the rights of research participants and the confidentiality of the collected data. Informed consent was obtained from all participants. Each of the parents consented to the participation of the adolescent child in the study. Without this consent, the minor participant did not receive access to the questionnaire.

The questionnaire was implemented via Google and was active at the end of October and the beginning of November 2021. At the time we conducted this research, physical distancing in public places was recommended in Poland, but not mandated. It was obligatory to wear preventive masks in public spaces. All age groups were also included in the voluntary vaccination programme.

The questionnaire consisted of 11 questions (Table 3). The questions concerned preventive health behaviours during the COVID-19 pandemic. The main outcome in this text was the relationship between parents’ and teenagers’ preventive health behaviours during the COVID-19 outbreak in terms of vaccination against COVID-19 and attitudes towards vaccination against COVID-19, as well as vaccination intention (in the case of unvaccinated people); wearing protective masks where it is compulsory and where it is not obligatory; maintaining physical distance; and disinfecting hands in public places. The sociodemographic factors were gender of the parent, gender of the child, the age of the parent, the age of the child and the place of residence.

### 2.3. Data Analysis

The collected data were statistically analysed using the Statistica version 13.3 software package (TIBCO Software Inc: Palo Alto, CA, USA) for advanced statistical data analysis. Descriptive statistics and correlation for non-parametric data (Spearman’s correlation) were used. Additionally, a Microsoft Excel (Microsoft Corporation: Redmond, Washington, DC, USA) spreadsheet for Windows was used to create charts and tables.

## 3. Results

### 3.1. Getting Vaccinated against COVID-19

More than 8 in 10 parents and 7 in 10 children declared that they had been vaccinated against COVID-19 (Table 4). 

There is a relationship between the answers of parents and adolescents. The correlation between the data shows a significant relationship (rs = 0.59). Pupils get vaccinated above all if their parents are also vaccinated, and the other way around: if their parents are not vaccinated, adolescents are not vaccinated (Table 4). There is a stronger correlation between mothers and daughters (r = 0.62) than between mothers and sons (r = 0.45). Parents with a secondary education degree had a higher response for the parent–child correlation (r = 0.55) than parents with a university degree (r = 0.46). Considering the place of residence of the parent–child dyad, a high correlation was achieved among urban residents (r = 0.66), moderate described parent–child dyads who stated place of residence as a village (r = 0.55). Inhabitants of medium-sized cities showed a low correlation (r = 0.36). A high correlation was observed when the parent was between 51 and 60 years of age (r = 0.68). Moderate correlation was observed for parents aged 41–50 years (r = 0.52) or 34–40 years (r = 0.46).

### 3.2. Attitudes toward Vaccination against COVID-19

Negative and rather negative attitudes towards vaccination against COVID-19 were reported by 1 in 10 parents and 2 in 10 students (Table 5).

There is a link between the attitudes of parents and their children about getting vaccinated against COVID-19. This correlation is important (rs = 0.52). Students showed positive or rather positive attitudes towards vaccination against COVID-19 when their parents held similar attitudes (Table 5). The strongest correlation was between fathers and sons (r = 0.76). A moderate correlation was observed between mothers and daughters (r = 0.57) and mothers and sons (r = 0.57). A high correlation was achieved for parents with secondary education (r = 0.62) and a moderate correlation for parents with primary education (r = 0.53). The highest correlation was achieved by those who declared their residence as a village (r = 0.68), although it was similar for people living in large cities (r = 0.62). A moderate correlation was observed among parent–child pairs who reported living in medium-sized cities (r = 0.46). A high correlation was observed for parents aged between 51 and 60 years (r = 0.61) and 41 to 50 years (r = 0.6). Decreasing parental age led to only a moderate correlation in the age groups (34 to 40 years) (r = 0.53).

### 3.3. Vaccination against COVID-19 Intention

Only 7% of the parents who have not been vaccinated (total: 27) and 5% of unvaccinated students (total: 52) planned to be vaccinated (Table 6).

As with the previous questions, we found a correlation between the intention of adults and their children to be vaccinated against COVID-19 (rs = 0.67). Non-vaccinated pupils in secondary education were more likely to be unwilling to change this situation if their parents were unwilling to do so (Table 6). A very high correlation between mothers and daughters was observed (r = 0.82), as well as a high correlation between mothers and sons (r = 0.69). Only a moderate correlation for the answers of adolescent children was observed for the parent group aged 41 to 50 years (r = 0.59). The highest correlation was observed among parents with a university degree (r = 0.68), as well as a slightly lower correlation among those with a secondary school degree (r = 0.66). Considering the variables of residence, rural-dwelling parents and children answers showed a significant correlation (r = 0.61).

### 3.4. Other Preventive Health Behaviours during COVID-19 Outbreak

#### 3.4.1. Wearing Protective Masks

Nearly 9 of the 10 parents reported wearing masks when required, while 76% adults chose the most positive answer, compared with only 50% of adolescents (Table 7).

When mask wearing was not obligatory, the rate was much lower, as only 15 in 100 parents and school students wore masks where it was not mandated (Table 8).

There was a statistically significant relationship between the wearing of protective masks in public spaces by parents and their teenage children. This applied to both compulsory and non-compulsory venues. Adolescents were more likely to wear protective masks in public spaces if their parents also did so. The correlation between the data shows a significant relationship in both cases (rs = 0.3 for compulsory places; rs = 0.42 for optional places). More detailed statistics on the answers to the question about wearing protective masks in compulsory places show a low correlation between mothers and daughters (r = 0.36) and between mothers and sons (r = 0.28). When looking at parental age, only a significant correlation between parents in the 41–50 age group and their children was observed (r = 0.33). A moderate correlation was observed in the child–parent dyad when the parent has a secondary education diploma (r = 0.55). The highest correlation appeared for place of residence in large city-dwelling pairs (r = 0.37). For cases of wearing masks in non-compulsory places, the highest correlation was observed between fathers and sons (r = 0.72). A moderate correlation was observed between mothers and daughters (r = 0.48) and a low correlation between mothers and sons (r = 0.39). In the parental age group, moderate correlations were observed for parents aged 34–40 years (r = 0.47) and for parents aged 41–50 years (r = 0.42). A moderate correlation was also observed among parents with a secondary education (r = 0.5), but this was low among parents with a primary education (r = 0.39). In the statistics that include place of residence, a moderate correlation was observed between large city-dwellers (r = 0.45) and parent–child pairs from medium-sized cities (r = 0.46). In the villages, the correlation was low (r = 0.35).

#### 3.4.2. Physical Distancing in Public Places

The legal guardians of children tend to maintain physical distance in public, although only 2 in 10 adults answered this question clearly positively. Only 1 of 10 adolescents fully positively declared keeping distance in public spaces (Table 9).

Secondary school pupils maintained physical distancing if their parents did so. The correlation between the data showed a significant relationship (rs = 0.42). Moderate correlation was observed between mothers and daughters (r = 0.55), while the lowest correlation was observed between mothers and sons (r = 0.28). Moderate correlation was observed in pairs when parents were aged 41–50 years (r = 0.45), while there was a low correlation for parents aged 34–40 years (r = 0.32). A high correlation was observed in pairs for parent with a secondary education (r = 0.63), while a low correlation was observed for parents with a primary education (r = 0.27).

#### 3.4.3. Disinfecting Hands in Public Places

Almost half of the adults strongly declared disinfecting hands in public places. For teenagers, it was just over 20% (Table 10).

There is a relationship between parents and children for disinfecting hands in public places (rs = 0.44). Secondary school pupils disinfected their hands in public places when their parents did so. A moderate correlation was observed between mothers and daughters (r = 0.52), while there was a low correlation between mothers and sons (r = 0.38). The highest correlation was observed for parents aged 51–60 years (r = 0.7) and their children, and there was a moderate correlation for parents aged 34–40 years (r = 0.46). Statistics on parental education showed moderate correlations between the answers of children and parents with higher education (r = 0.42) and secondary education (r = 0.49). A moderate correlation was also observed for pairs from large cities (r = 0.44) and small cities (r = 0.52). The correlation was low among villagers (r = 0.36).

## 4. Discussion

The main research question concerned the relationship between parents’ and teenagers’ preventive health behaviours during the COVID-19 outbreak and its conditions. Regarding all the variables distinguished in this study, these relationships between parental and adolescent answers are statistically significant. Preventive actions taken by parents and a positive attitude towards restrictions and obligations were factors related to adolescents’ behaviours and attitudes. The relationship between the behaviour of parents and children is nothing new, including in the context of health behaviours [24,25,26,27,28,29,34], but in our study it concerns the situation of a pandemic and is interpreted as an element of HBM, which may be a starting point for further research.

It is worth mentioning that the declarations of the adults indicate that they followed the recommendations more often than their children did, and that they tended to have a more positive attitude towards restrictions. This is consistent with the results of other diagnoses and studies. Young people are a group that, in the case of the COVID-19 pandemic, has been less active in preventive actions than adults [19,20,21]. The results of other studies show a significant decrease in the physical well-being of children and adolescents during the COVID-19 pandemic [35,36,37,38]. Undoubtedly, it is associated with an observable decrease in physical activity, a marked increase in emotional disorders and problems, and a level of stress that can cause psychosomatic symptoms [39]. Therefore, despite less anxiety related to the physical aspects of possible illness, they still require a dedicated preventive programme because of the threat to psychological well-being. Considering our and other studies’ results [40], parents could be involved in this kind of preventive programmes as role models for children. Their awareness of the role played by their guidance to action should be increased. Self-efficacy and participation in caring for others can contribute to increased well-being. In light of our research, there is a significant role of parental influence in this process.

Other studies also indicate that other environmental influences are also important, especially those close to the everyday life of young people, such as the media and social media [41,42]. As concluded by Hamilton et al. [42], social media provides essential tools for teens’ access to COVID-related resources, autonomy and identity exploration, creative expression, and social connection. Considering the results of our research, the role of social media should be noticed by parents and included in their activities with children.

The relationship between the health behaviours of children and their parents is more evident in the case of fathers than of mothers, for parent–child dyads from big cities and for parents without higher education qualifications.

When interpreting the results, the fact that the research group of parents was primarily composed of mothers is important. The questionnaire reached parents, regardless of their gender, but it turned out that it was mainly completed by women. The observed tendency could be the result of the dominant trend in Poland, according to which women more often than men are responsible for taking care of children at home, including taking care of school-related matters (the questionnaire was provided by schools) [43]. However, this result could also be interpreted differently. Other studies have shown that women more often take various preventive health actions [44]. For example, in the study by Kamran et al. [45] conducted during the COVID-19 outbreak, it was shown that women were characterized by a better attitude towards the restrictions than men. This is despite the fact that men generally experience a more severe course of coronavirus infection and are at the greater risk of death [46]. In light of these results, it can be assumed that the overrepresentation of women in the sample may be related not only to the fact that they deal with matters related to children’s education, but also to their involvement in the education of children in the field of health prevention. The fact of filling in the questionnaire and encouraging the child to do so is a cue to action. Other studies have indicated that it is mothers who participate in preventive programmes addressed to parents [47]. Interestingly, although other studies also show the same results—and mothers’ support is a stronger factor protecting adolescents from engaging in risky behaviours than fathers’ support [48]—in our study, there is a stronger correlation between preventive health actions taken by fathers and children than between mothers and children. Thus, although considerably fewer men than women took part in the study, their results in terms of the relationship between their behaviour and that of their children were highly significant, and more significant than in the case of mothers. As Trumello et al. [49] concluded in their research on father-child relationships during the first peak of the COVID-19 outbreak, there is a need to consider the effects of the pandemic on fathers, as they are overlooked by research mainly focused on mothers. Fathers’ role in the negative consequences of the COVID-19 outbreak for children and adolescents’ development and in preventive action promotion is worth considering. Planning specific interventions should take fathers into account, especially in times of crisis, such as the pandemic, because this situation changed, e.g., the parental division of the household [50] giving a chance to involve fathers more in childcare and home-schooling activities. Findings from our research indicate that the fathers’ impact on children’s decisions can be significant; hence, strengthening involvement in everyday family matters can be used positively in the case of preventive health behaviours taken by the family members.

When it comes to the strength of the correlations, parent–child pairs from large cities and villages and those in which the parent has a secondary or primary level of education stood out. Previous studies have shown that a high level of education among parents is one of the determinants of a fully negative attitude towards vaccinations [51]. Additionally, having parents with a higher level of education is related to the willingness to express one’s own opinions and make one’s own decisions [52], which may be a reason for choosing behaviours other than those of the parents during the COVID-19 outbreak.

## 5. Conclusions

According to the HBM, behaviour-changing interventions are most effective when they relate to the individual’s specific perceptions of vulnerability, benefits, barriers and self-efficacy. Interventions focusing on this model may include anticipating risks for specific groups and individual ways of changing beliefs. Young people who are not convinced of their own vulnerability and are overburdened in the event of a pandemic engage in less preventive behaviour than adults, but it turns out that the parental role model is an effective guideline. Our recommendations, therefore, include educating parents about their influence on children’s behaviour, particularly in difficult situations, which undoubtedly include pandemics.

When interpreting the results regarding the stronger influence of the father or education and place of residence, it is worth remembering that a strong correlation may apply to both consistent health-related choices and consistent risky choices (for example, high convergence in not wearing protective masks). Therefore, the lack of consistency may indicate not following the parent, who takes risky behaviours. Our recommendations do not refer to the privileging of, for example, parents from large cities, but to the use of knowledge among parents in prevention programmes that affect the behaviour of children. The conclusions from our research, which can be used in preventive programmes, show that there is more consistency in the behaviours of children (especially sons) and fathers, and children and parents with lower and secondary education, as well as from large cities and villages, thus these people tend to have the greatest impact on their own children.

## 6. Limitations

This study was not experimental; however, it indicates a relationship between the behaviour of parents and children. We treated this relationship in terms of the HBM (as a clue-to-action). We assumed that in the case of a pandemic, young people do not meet some of the assumptions of the model, such as perceiving the health risks as serious, feeling personally vulnerable and expecting to have more significant benefits than costs, hence in their case, getting cues-to-action can be an important trigger to protect themselves and others. Further research using the HBM on youth health behaviours should be to confirm our results and look for ways to use clue-to-action insights.

The limitations of the study also include the small proportion of men in the sample. This does not allow for generalizations regarding the role of fathers, except that this is a group that should be particularly included in programmes addressed to parents, indicating their role in the health behaviours undertaken by children, because they do not even participate in research related to the topic of preventive health behaviours.

## Figures and Tables

**Table 1:** Socio-demographic characteristics of the students.

Gender	N (%)
Female	82 (40.80)
Male	115 (57.20)
Other	2 (1.00)
Did not want to specify	2 (1.00)
Age	N (%)
14	23 (11.44)
15	79 (39.3)
16	54 (26.87)
17	45 (22.39)
Place of residence	N (%)
Countryside	68 (33.83)
Small town	76 (37.81)
Large city	57 (28.36)

**Table 2:** Socio-demographic characteristics of parents.

Gender	N (%)
Female	187 (93.03)
Male	14 (6.97)
Other	0 (0.00)
Did not want to specify	0 (0.00)
Age	N (%)
34–40	40 (19.90)
41–50	135 (67.16)
51–60	25 (12.44)
61 and more	1 (0.50)
Place of residence	N (%)
Countryside	71 (35.32)
Small town	72 (33.82)
Large city	58 (28.86)

**Table 3:** Questionnaire content.

Age (Open Question)
Gender Female Male Other Did not want to specify
Place of residence Countryside Small town Large city
Education (only parents)
What is your attitude towards the COVID-19 vaccination? Positive Rather positive Rather negative Negative
Have you been vaccinated against COVID-19? Yes No
Are you going to get vaccinated against COVID-19? (Questions for unvaccinated people) Yes Perhaps yes Perhaps not No
Do you wear a protective mask in places where it is compulsory? Yes Generally yes Generally no No
Do you wear a protective mask where it is not compulsory? Yes Generally yes Generally no No
Do you disinfect your hands when you are in public places (e.g., shops, shopping mall)? Yes Generally yes Generally no No
Do you keep your distance (1.5–2 m) in public places (e.g., shops, shopping mall, school)? Yes Generally yes Generally no No

**Table 4:** COVID-19 vaccination status.

	No [N (%)]	Yes [N (%)]
Parents	27 (13.43)	174 (86.57)
Students	52 (25.87)	149 (74.13)

**Table 5:** Attitudes towards vaccination against COVID-19.

	Negative [N (%)]	Rather Negative [N (%)]	Rather Positive [N (%)]	Positive [N (%)]
Parents	13 (6.47)	12 (5.97)	32 (15.92)	144 (71.64)
Students	16 (7.96)	22 (10.95)	45 (22.39)	118 (58.71)

**Table 6:** Vaccination against COVID-19 intention.

	No [N (%)]	Generally No [N (%)]	Generally Yes [N (%)]	Yes [N (%)]
Parents	13 (48.15)	7 (25.92)	5 (18.52)	2 (7.40)
Students	16 (30.77)	17 (32.70)	14 (26.92)	5 (9.61)

**Table 7:** Wearing of protective masks when obligatory.

	No [N (%)]	Generally No [N (%)]	Generally Yes [N (%)]	Yes [N (%)]
Parents	5 (2.49)	5 (2.49)	37 (18.41)	154 (76.62)
Students	7 (3.48)	15 (7.46)	77 (38.31)	102 (50.75)

**Table 8:** Wearing of protective masks when non-obligatory.

	No [N (%)]	Generally No [N (%)]	Generally Yes [N (%)]	Yes [N (%)]
Parents	99 (49.25)	72 (35.82)	17 (8.46)	13 (6.47)
Students	126 (62.69)	46 (22.89)	19 (9.45)	10 (4.98)

**Table 9:** Physical distancing in public places.

	No [N (%)]	Generally No [N (%)]	Generally Yes [N (%)]	Yes [N (%)]
Parents	13 (6.47)	31 (15.42)	116 (57.71)	41 (20.4)
Students	45 (22.39)	50 (24.88)	80 (39.80)	26 (12.94)

**Table 10:** Disinfecting hands in public places.

	No [N (%)]	Generally No [N (%)]	Generally Yes [N (%)]	Yes [N (%)]
Parents	14 (6.97)	27 (13.43)	71 (35.32)	89 (44.28)
Students	42 (20.90)	29 (14.43)	86 (42.79)	44 (21.90)

## Data Availability

The data presented in this study are available on request from the authors who carried out the analyses (M.K and N.B.). The data are not publicly available due to information that could compromise the anonymity of the research participants.
